# The Visceral Adipose Index in Relation to Incidence of Hypertension in Chinese Adults: China Health and Nutrition Survey (CHNS)

**DOI:** 10.3390/nu12030805

**Published:** 2020-03-18

**Authors:** Yong Xue, Qun Shen, Chang Li, Zijian Dai, Tingchao He

**Affiliations:** 1 Beijing Advanced Innovation Center for Food Nutrition and Human Health, Key Laboratory of Plant Protein and Grain Processing, National Engineering and Technology Research Center for Fruits and Vegetables, College of Food Science and Nutritional Engineering, China Agricultural University, Beijing 100083, China; xueyong@cau.edu.cn (Y.X.); chang_210@126.com (C.L.); daizijian666@163.com (Z.D.); 2 Key Laboratory of Plant Protein and Grain Processing, National Engineering and Technology Research Center for Fruits and Vegetables, College of Food Science and Nutritional Engineering, China Agricultural University, Beijing 100083, China; shenqun@cau.edu.cn; 3 Department of Nutrition and Food Hygiene, School of Public Health, Peking University, No. 38, Xueyuan Road, Haidian District, Beijing 100191, China

**Keywords:** visceral adipose index, hypertension, abdominal obesity, visceral adipose index, The China Health and Nutrition Survey, population-based cohort study

## Abstract

Hypertension is the most crucial single contributor to global burden of disease and mortality, while weight loss as a non-pharmacological strategy is recommended to reduce blood pressure. This study aims to examine the association between visceral adipose index (VAI) and hypertension in Chinese adults. Data were collected from the China Health and Nutrition Survey (CHNS), consisting of 8374 apparently healthy participants aged ≥18 years in the 2009 CHNS for cross-sectional analysis, and 4275 participants at entry from 2009 to 2011 for cohort analysis. Height, weight, waist circumference, blood pressure (BP), and blood lipid were measured. Information of population characteristics, smoking status, alcohol consumption, physical activity, and diet were determined by validated questionnaire. Higher VAI scores were significantly associated with higher BP levels and higher risk of hypertension after adjustment with potential confounders (all *p*-trend < 0.001). The adjusted hazard ratio of hypertension was 1.526 (95%CI: 1.194, 1.952; *p*-trend < 0.01) for participants in the highest quartile of VAI scores when compared with those in the lowest quartile after adjustment for age, physical activity, antihypertensive medication, total energy intake, salt intake, and other major lifestyle factors. VAI scores were significantly, longitudinally associated with hypertension development among apparently healthy Chinese adults.

## 1. Introduction

Hypertension is known as one of the three crucial risk factors (smoking, hypertension, and overweight) shared by all non-communicable diseases (NCDs) [1], and also the leading single contributor to disease burden and death worldwide [2,3]. Hypertension is also an independent disease and in this respect differs from the two others that are more clearly related to individual behavior and decisions. In particular, elevated blood pressure (BP) was reported to be consistently associated with several cardiovascular diseases (CVDs) such as coronary heart disease (CHD), ischemic heart disease (IHD), stroke, and myocardial infarction [2]. During the past two decades, the global prevalence of hypertension has rapidly increased from 26.4% [4] to 31.1% [5] in adult population, and what even worse was that prehypertension affected 25–50% adult population [6], predicating enormous reserves of hypertension patients. As a highly modifiable risk factor of CVDs [7], the prevention and management of hypertension are major public health challenge worldwide to be addressed. 

The associations of overweight/obesity and some other adiposity indicators with the risk of hypertension were previously reported in substantial cross-sectional and time-series studies [8,9,10,11], and thus losing weight, as one of the non-pharmacological strategies, is commonly recommended to lower BP [3]. Several simple and low-cost adiposity indicators were developed including general adiposity index (body mass index (BMI) and height-adjusted body weight) and central adiposity indicators such as waist circumference (WC), hip circumference, waist-to-hip ratio, waist-to-height ratio, body adiposity index (BAI), and visceral adiposity index (VAI) [7,11,12]. Though BMI and WC are commonly measured in current research, BMI in some cases is not a suitable predictor for the percentage of body adipose [13] and WC is unable to distinguish visceral from subcutaneous fat [14]. Instead, VAI was proposed as a reliable and comprehensive indicator of determining visceral adipose associated with cardiometabolic status, since VAI comprehensively takes anthropometric and metabolic factors into account and was reported to be associated with visceral adipose tissue area and volume, but not with subcutaneous adipose tissue [14]. Therefore, VAI was introduced to be a surrogate indicator of adipose tissue function, which can more directly predict the progress and risk of CVDs [12,15].

There were substantial researches exploring the associations of VAI with chronic diseases in human, which proved that VAI was negatively associated with insulin sensitivity [16,17] and identified as a powerful indicator for pre-diabetes or diabetes [18,19,20], and positively associated with the risk of hyperuricemia [21] and nonalcoholic fatty liver disease (NAFLD) [22]. Additionally, the results of several epidemiological studies [9,15,23] exploring the association between visceral adiposity and risk of hypertension showed that VAI was positively associated with the risk of hypertension in Chinese adult population [9] or Japanese Americans [24], and baseline scores of VAI could predict hypertension and CVD incident in prehypertension or healthy population [15,23], whereas others reported no association [25] or an association in women but not men [26]. Moreover, there has been no large population-based cohort study in China on the relationship between VAI and risk of hypertension. Therefore, this study aims to explore the association between VAI and the risk of hypertension in a large population-based, across-sectional study from the 2009 phase of the China Health and Nutrition Survey (CHNS), and to estimate the effect of VAI on hypertension progression of healthy adult population in the CHNS from 2009 to 2011.

## 2. Materials and Methods 

### 2.1. Study Design and Participants

**Figure 1:**
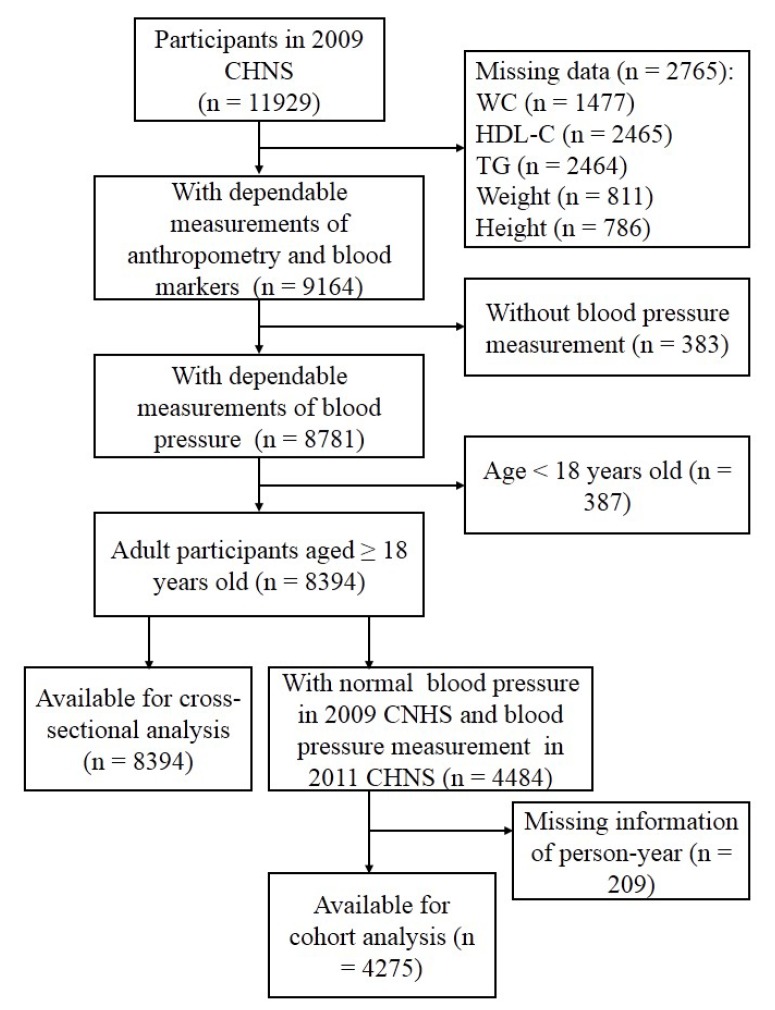
Participant flow diagram. CHNS, the China Health and Nutrition Survey; WC, waist circumference; HDL-C, high-density lipoprotein cholesterol; TG, total triglycerides.

The CHNS is an ongoing open prospective, population-based cohort study, which was administrated in nine provinces in China (vary substantially in geography, economic development, and health status) between 1989 and 2011 [27,28]. The CHNS was designed to explore the effect of social and economic transformation of Chinese society on nutritional and health status of Chinese population. Since 1989, nine waves of data collection (e.g., 1989, 1991, 1993, 1997, 2000, 2004, 2006, 2009, and 2011) have been implemented, whereas the blood samples were only collected in the 2009 CHNS. According to the 2010 census, the nine provinces included in the CHNS constituted 47% of China’s population [27] and the scientific rationale and design of the CHNS have been described in detail elsewhere [29,30]. In brief, a multistage, random-cluster sampling process was performed to select samples in a large higher-income city, a lower-income city, and four counties (1 high-, 2 middle-, and 1 low-income according to per-capita income provided by the National Bureau of Statics) within each province. Urban and suburban neighborhoods within the cities and villages and townships within the counties were selected randomly as the primary sampling unit, in which twenty households were randomly selected and all individuals were surveyed. Considering the fact that blood lipids, as the core indicators of VAI, were only detected in the 2009 CHNS, this paper used the cross-sectional data collected from the 2009 CHNS as the baseline information. Total of 11929 apparently healthy participants attended the baseline visit, during which they gave their informed consent and completed a structured questionnaire that asked about socioeconomic characteristics, lifestyle exposures (including diet, alcohol consumption, smoking status, and physical activity), general health, and medical history. All participants also completed physical measurements and provided blood samples. In this study, we excluded 2765 participants without measuring height, weight, WC, total triglycerides (TG), or high-density lipoprotein cholesterol (HDL-C), 383 without BP measurement, and 387 aged less than 18 years, resulting in 8394 adults in the baseline analysis. Of these participants, 4484 with normal BP in 2009 were measured again in the 2011 CHNS. Finally, the participants (*n* = 4275) who had person-year information were included in the cohort analysis (Figure 1). The data of CHNS are publicly available, and the researchers from the National Institute for Nutrition and Health, Chinese Center for Disease Control and Prevention and the Carolina Population Center, University of North Carolina at Chapel Hill had received ethic approval in the Institute Review Board.

### 2.2. Anthropometric Measurements and Serum Biochemical Parameters

Following standard procedures, anthropometric measurements were administrated by well-trained researchers in private and comfortable room. All participants were requested to remove bulky clothing and shoes before measurement. Standing height was measured to the nearest 0.1 cm using a SECA 206 wall-mounted metal tape, and weight was measured to the nearest 0.1 kg using a calibrated beam balance. WC was measured to the nearest 0.1 cm using a Seca tape measure (Seca north America, Chino, CA, USA). BMI (kg/m^2^) was calculated as weight in kilograms divided by height in meters square. Triplicate measurements of BP were taken after 5 min at sitting and with at least 1 min between recordings with the use of mercury sphygmomanometer, and means of the three BP measurements were used in the final analysis. Participants who met at least one of the following criteria: (1) systolic BP (SBP) ≥140mmHg, (2) diastolic BP (DBP) ≥90mmHg, (3) ever diagnosed with hypertension by a physician were defined as hypertension patients. 

According to a standard protocol, blood samples (12 mL) were collected via venipuncture after at least 8 h of overnight fasting, and then transferred to the local hospital for further treatment within 2 h of collection. The blood samples in red-stoppered tube (4 mL) were centrifuged at 3000 × *g* for 15 min at room temperature; serum samples were frozen and stored at −86 °C for the subsequent laboratory analysis. According to strict quality control standards, all samples were verified and analyzed in a national central laboratory in Beijing (Medical laboratory accreditation certificate: ISO 15189:2007) [31]. TG in serum was analyzed by the glycerol-3-phosphate oxidase-phenol and aminophenazone GPO-PAP method, HDL-C and low-density lipoprotein cholesterol (LDL-C) by enzymatic method, total cholesterol (TC) by the cholesterol oxidase-phenol and aminophenazone (CHOD-PAP) method, and fasting blood glucose by glucose oxidase-phenol and aminophenazone (GOD-PAP) method. All biochemical assessment aforementioned were performed using Hitachi 7600 automated analyzer (Hitachi Inc., Tokyo, Japan) [32]. VAI score was calculated by pervious reported formula [14]:

For men,

(1) $$\mathrm{VAI}=\left(\frac{\mathrm{WC}}{39.68+1.88\times \mathrm{BMI}}\right)\times (\frac{\mathrm{TG}}{1.03})\times (\frac{1.31}{\mathrm{HDL}})$$

For women,

(2) $$\mathrm{VAI}=\left(\frac{\mathrm{WC}}{36.58+1.89\times \mathrm{BMI}}\right)\times (\frac{\mathrm{TG}}{0.81})\times (\frac{1.52}{\mathrm{HDL}})$$

### 2.3. Physical Activity, Dietary intake, and Other Covariates

Information of physical activities in the past 7 days categorized as four groups (domestic, occupational, leisure, and transformation physical activity) were collected using staff-administered questionnaires, as described in detail elsewhere [33]. The level of physical activity was estimated using metabolic equivalent (MET)-hours-per-week, which was summarized by multiplication of time spent in each activity and specific MET value [34]. The dietary assessment in the CHNS was performed by well-trained investigators via a combination of three consecutive 24-h recalls at the individual level and a food inventory at the household level over the same period (2 weekdays and 1 weekend day). Detailed information of the dietary assessment has been published previously [29,35]. In brief, individual dietary intake was estimated by asking each participant to report information of all food items (including the amounts, types of meal, and place of consumption) and the proportion of each dish during the previous day, with the aid of food pictures and models. Household food consumption was estimated by weighting household food inventory and the wastage. For each dish prepared at home, the amount of food consumed by each participant was estimated based on household food consumption and the proportion of individual consumption. Dietary intake of total energy was calculated by CHNS on the basis of the Chinese Food Composition Table (2002 and 2004) and the individual salt intake (g/day) was estimated based on the change of salt inventory at household level and the proportion of each individual’s energy intake. Furthermore, dietary intake of energy was categorized into four groups according to their quartiles to reflect none, low, medium, and high intake and salt intake into three groups according to its tertiles. Other covariate factors such as countryside, age, gender, nationality (Han or others), educational status, alcohol consumption (yes or no), smoking (current or not current), usage of antihypertensive medications were enquired and recorded by well-trained researchers using structured questionnaire.

### 2.4. Statistical Analysis

VAI was classified by the quartiles in the corresponding populations. Descriptive analyses estimated the mean ± standard deviations (SDs) or median (interquartile ranges, IQRs), as applicable, or number (frequency) of the discrete covariates within each category of VAI with statistical significance estimated using ANOVA or Kruskal-Wallis test or Chi-square test, respectively. To explore the association of VAI with BP levels and risk of hypertension in the 2009 CHNS, we constructed general linear regression models (SBP and DBP levels) and logistic regression models (risk of hypertension) with replicated analyses: original model without any adjustments (model 1), model 2 adjusted for socioeconomic factors (countryside, age, gender, nationality, and education), model 3 additionally adjusted for lifestyle factors (smoking status, alcohol consumption, physical activity, and usage of antihypertensive medication) and dietary factors (total energy and salt intake), model 4 further adjusted for TC levels. β Regression coefficients and 95% confidence intervals (CIs) in the linear regression and odds ratios (ORs) and 95% CIs in the logistic regression were estimated for BP and risk of hypertension, respectively. Furthermore, multivariable Cox proportional hazards were applied to determine the hazard ratios (HRs) and 95% CI of hypertension for each quartile of the VAI scores, with the lowest quartile (Q1) always used as the reference. All statistical tests were two-sided tests and were carried out using the Statistical Package for Social Sciences Version 24.0 (SPSS Inc., Chicago, IL, USA). *p* < 0.05 was considered to be statistically significant.

## 3. Results

### 3.1. Characteristics of the Subjects from the 2009 CHNS

In the present study, 8394 participants aged 18–99 years old from the 2009 CHNS were included in cross-sectional analysis and 4275 participants who participated in both the 2009 and 2011 CHNS were included in cohort analysis (Figure 1). Population characteristics of male and female participants are shown in Table 1 and Table 2, respectively. Among the male subjects (*n* = 3911), there is no significant differences of educational level, total energy intake, salt intake, smoking and drinking habits among the subgroups according to the VAI scores (all *p* > 0.05). However, compared with the participants who had lower VAI scores, those with higher VAI scores were less likely to reside in countryside, be older, have higher concentrations of HDL-C, and have higher levels of physical activities, whereas were more likely to have higher BMI, WC, SBP, and DBP, higher concentrations of FBG, TG, TC, and LDL-C (all *p* < 0.05), receive medications for hypertension. Similar characteristics were also found in female participants (*n* = 4483) with higher VAI scores except for living location, age, educational level, and drinking habit when comparing with those with lower VAI scores. The association of VAI scores with living location was not found in female participants. Meanwhile, positive association of age and negative association of educational level and alcohol consumption with the VAI scores were found in female participants.

### 3.2. Associations of VAI with Blood Pressure Levels in the 2009 CHNS

The levels of VAI scores were significantly, positively associated with SBP and DBP levels in a dose-response manner (both ***p***-trend < 0.001) after progressive adjustment for potential confounders (Table 3) including countryside, age, gender, nationality, education, smoking status, alcohol consumption, physical activity, antihypertensive medication, total energy intake, and salt intake. Those dose-response associations were not markedly changed when further adjusted for levels of TC. Compared with participants who had the lowest quartile of VAI scores, those with the highest quartile showed significantly higher levels of SBP (by 5.197 mmHg, 95% CI: 4.196, 6.199; *p*-trend < 0.001) and DBP (by 3.978 mmHg, 95% CI: 3.321, 4.634; *p*-trend < 0.001). We further performed the cross-sectional analyses stratified by gender and the results showed positive associations between levels of VAI scores and BP (including SBP and DBP) levels in both male and female participants (all *p*-trend < 0.001). Compared with male participants who had the lowest quartile of VAI scores, those with highest showed significantly higher levels of SBP (by 3.315 mmHg, 95% CI: 1.889, 4.740; *p*-trend < 0.001) and DBP (by 3.516 mmHg, 95% CI: 2.546, 4.487; *p*-trend < 0.001). Similarly, for women, compared with the reference group (Q1), those participants with the highest quartile of VAI scores showed significantly higher levels of SBP (by 6.157 mmHg, 95% CI: 4.737, 7.577; *p*-trend < 0.001) and DBP (by 4.409 mmHg, 95% CI: 3.144, 4.954; *p*-trend < 0.001).

### 3.3. Associations of VAI with Risk of Hypertension in the 2009 CHNS

We further applied multivariable-adjusted logistic regression models to examine the association of VAI with the prevalence of hypertension. Our results showed that the levels of VAI scores were significantly, positively associated with the prevalence of hypertension in a dose-response manner (all *p*-trend < 0.001) after adjustment for potential confounders (Table 4). Those dose-response associations did not change when further adjusted for levels of TC, or when restricted to men or women. Compared with participant with the lowest quartile of VAI scores, those with the highest quartile had significantly increased risk of hypertension (OR: 2.299; 95% CI: 1.939, 2.726; *p*-trend < 0.001). In male participants, those with the highest quartile of VAI scores had significantly higher risk of hypertension when compared to the reference group (OR: 1.849; 95% CI: 1.467, 2.329; *p*-trend < 0.001); similarly, this association was also found in female participants (OR: 2.781; 95% CI: 2.141, 3.612; *p*-trend < 0.001). 

### 3.4. Associations of VAI with Incidence of Hypertension from 2009 to 2011

To examine the association between VAI scores and incidence of hypertension, we excluded participants who was hypertensive at baseline and who had no follow-up data. A total of 587 cases of incident hypertension were documented among 4275 participants with normal BP at baseline. The levels of VAI scores were significantly, positively associated with the incidence of hypertension in a dose-response manner (*p*-trend = 0.005). Compared with those who were in the lowest quartile of VAI scores, participants in the highest quartile were 52.6% more likely to develop hypertension (HR: 1.526; 95% CI: 1.194, 1.952; *p*-trend = 0.005) independent of baseline BP and potential confounders (Table 5). Though significant, positive association between VAI scores and the incidence of hypertension was found in total and male participants (both *p*-trend < 0.05), this association was not found in female participants (*p*-trend > 0.05) after adjustment for potential confounders. However, compared with those who were in the lowest quartile of VAI scores, female participants in the highest quartile were 49.8% more likely to develop hypertension (HR: 1.498; 95% CI: 1.041, 2.157; *p* < 0.05).

## 4. Discussion

To our knowledge, this is the first cohort study based on a large-scale, diverse population in China to have examined the associations between VAI scores and BP levels as well as risk of hypertension. In cross-sectional analyses of 8394 adult participants in 2009 CHNS, our results showed that the higher VAI scores were associated with higher levels of BP and risk of hypertension. Those positive associations were independent of socioeconomic, medication, and lifestyle factors frequently associated with VAI scores and hypertension, such as countryside, age, gender, nationality, education, smoking status, alcohol consumption, physical activity, antihypertensive medication, and dietary intake of total energy and salt. Further analysis of these relations in subgroups stratified by gender showed that both male and female participants with higher VAI scores had significantly higher levels of BP and risk of hypertension. When we further confirmed those relationships in cohort analysis, our results showed that the VAI scores were positively, significantly associated with the incidence of hypertension, especially for male participants. 

Several epidemiological studies explored the association between VAI scores and risk of hypertension or CVDs but with inconsistent conclusions on the predictive ability of the VAI [10,11,15,25,26,36]. Though the positive association between the VAI scores and the risk of hypertension were demonstrated in different populations, there are still considerable studies that reached differing conclusions. One cross-sectional analysis in 111,911 Mexican adults showed that central adiposity makers are less strong predicators of BP compared with the markers of general adiposity such as overweight or obese [7]. Janghorbani et al. [36] assessed the incidence of hypertension in 1375 non-diabetic and non-hypertensive participants over a 7-year follow-up and reported that greater VAI scores weakly predicted hypertension whereas the hypertriglyceridemic waist was a stronger predictor. Similarly, another study with 1627 individuals in Brazil reported that the indicators of adiposity such as WC and BMI were better associated with hypertension when compared with BAI and VAI [11]. However, WC and BMI in some cases are not suitable predictors of the percentage of body adipose [13], and unable to distinguish between visceral and subcutaneous fat [14]. Hence, our priority was to explore the association between VAI scores and risk of hypertension.

Our study demonstrated significant, positive associations of VAI scores with BP and risk of hypertension in 2009 CHNS, and those independent associations remained stable when considering potential confounders including demographic characteristics, lifestyle habits, and intake of salt. Previous studies had reported that VAI was positively associated with the risk of prehypertension and hypertension incidence in Chinese participants [9,23]. Our study support that VAI scores may be an independent risk factor for hypertension progression in general population as well. Considering the fact that the intrinsic defect of the cross-sectional study would confine the exploration and analysis of the potential causal relationship, longitudinal analysis of 2009-2011 CHNS also been performed in this study. To our knowledge, it is the first large population-based cohort study in Chinese population focusing on the relationship between the VAI scores and incidence of hypertension, and the results indicated that higher VAI was associated with an increasing incidence of hypertension. After more than 10 years of follow-up, Hayashi et al. [24] found that intra-abdominal fat was an independent risk factor for hypertension incidence in Japanese Americans. Our study was consistent with their results, since it demonstrated increased risk of hypertension in the highest quartile of VAI scores among male and female participants. Previous studies have demonstrated that substantial risk factors of CVDs are responsible for hypertension, especially obesity. VAI was proposed as a reliable and comprehensive indicator of visceral adipose and its association with risk of CVD has been studied in other epidemiological studies. VAI had significant predictive of 10-year CVD incidence among male Caucasian/Mediterranean adults, and it showed better predictive effective than typically performed anthropometric measurements [15].

The details of the underlying mechanism about the effect of VAI on hypertension remains to be determined. VAI was proposed as a reliable and comprehensive indicator of determining visceral adipose [14], and was suggested to replace visceral computed tomography scanning as a marker for visceral adiposity [17]. Both central obesity and high BP are well-known indicators of metabolic syndrome, and the mechanisms of obesity and obesity-related hypertension are complicated and at times interdependent [37]. One prospective cohort study in Japanese Americans demonstrated that the incidence of hypertension is positively associated with accumulation of intra-abdominal adipose tissue, while not with thigh subcutaneous fat or abdominal subcutaneous fat [38], suggesting that location of adipose depot plays a crucial role in the progression of hypertension. Excessive adiposity tissue might be accompanied by the alterations in hormone, inflammation, and endothelia, which induces the succeeding events through increasing insulin resistance, stimulation of renin- sympathetic nervous system, angiotensin-aldosterone system, endothelial dysfunction and renal sodium reabsorption, ultimately causing high BP [37]. Relationship of visceral adipose tissue and risk of hypertension occurrence may vary in different countries and ethnic groups [24]. However, compared with dual-energy x-ray absorptiometry (DXA) and magnetic resonance imaging (MRI) methods, VAI can act as a simpler and more economical index to evaluate visceral adipose and the risk of hypertension. 

Strengths of the present study include the large nationwide, diverse population-based design, prospective follow-up design, and adjustment for main lifestyle factors. Except for some common weakness of any observational study and confounding from some unknown factors, some other limitations also need to be considered. One major concern is that the short follow-up period as well as loss to follow-up might would bias our results, although it is less likely that the cases of loss to follow-up are associated with both VAI scores and hypertension. Second, we cannot rule out the possibility that unmeasured factors or residual confounders might influence the associations observed in present study, even though numbers of confounders were adjusted. 

## 5. Conclusions

Our findings indicated that the VAI scores were significantly, positively associated with BP levels and the prevalence of hypertension in the Chinese adult population. Additional data from large cohort and animal studies may be very important to confirm these findings and to elucidate their underlying mechanisms. In order to prevent or delay the progression of hypertension and some other NCDs, substantial attention has been devoted to improve their potentially modifiable factors in recent years. Thus, further studies are warranted to determine whether reduction of VAI scores may improve the risk of hypertension, and thereby reduce the risk of diabetes, NAFLD, and CVDs.

## Figures and Tables

**Table 1:** Characteristics of the male subjects from the 2009 CHNS according to the quartiles of visceral adipose index (VAI) scores.

Variable	Q1 (<0.7339)	Q2 (0.7340–1.2613)	Q3 (1.2614–2.3037)	Q4 (≥2.3038)	Total	*p*
No. participants	978	978	977	978	3911	
Han nationality (%)	848 (86.7)	866 (88.5)	891 (91.2)	868 (88.8)	3473 (88.8)	0.018
Countryside (%)	697 (71.3)	694 (71.0)	637 (65.2)	612 (62.6)	2640 (67.5)	<0.001
Age (years)	51.9 ± 16.2	50.6 ± 15.6	50.3 ± 14.7	49.5 ± 13.9	50.6 ± 15.1	0.007
BMI (kg/m^2^)	21.4 ± 2.8	22.7 ± 3.0	23.9 ± 3.2	25.2 ± 3.4	23.3 ± 3.4	<0.001
Educational level						0.516
Middle school or below	935 (83.5)	706 (72.2)	662 (67.8)	635 (64.9)	2780 (71.1)	
High school	158 (14.1)	202 (20.7)	247 (25.3)	245 (25.1)	848 (21.7)	
College or above	27 (2.4)	70 (7.2)	68 (7.0)	98 (10.0)	283 (7.2)	
WC (cm)	78.4 ± 8.9	82.6 ± 9.2	86.3 ± 9.6	90.2 ± 9.3	84.4 ± 10.2	<0.001
SBP (mmHg)	124.6 ± 18.0	124.9 ± 17.3	126.4 ± 16.9	128.9 ± 17.8	126.2 ± 17.6	<0.001
DBP (mmHg)	80.2 ± 11.0	81.1 ± 11.1	82.5 ± 10.9	84.8 ± 11.0	82.2 ± 11.1	<0.001
FBG (mmol/L)	5.1 ± 0.9	5.2 ± 1.2	5.5 ± 1.5	6.2 ± 2.3	5.5 ± 1.6	<0.001
TG (mmol/L)	0.7 ± 0.3	1.1 ± 0.3	1.7 ± 0.4	3.8 ± 2.4	1.8 ± 1.7	<0.001
TC (mmol/L)	4.5 ± 0.8	4.7 ± 0.9	4.9 ± 1.0	5.1 ± 1.0	4.8 ± 1.0	<0.001
HDL-C (mmol/L)	1.8 ± 0.8	1.4 ± 0.3	1.3 ± 0.2	1.1 ± 0.2	1.4 ± 0.5	<0.001
LDL-C (mmol/L)	2.7 ± 0.8	3.0 ± 0.9	3.2 ± 1.0	2.8 ± 1.0	2.9 ± 1.0	<0.001
Total energy intake (kcal/day)						0.153
Median	2311.5	2300.3	2271.2	2248.8	2282.4	
IQR	1850.7–2796.4	1890.3–2739.6	1894.1–2683.8	1855.2–2660.5	1875.7–2721.5	
Salt intake (g/day)						0.646
Median	7.4	7.6	7.4	7.2	7.4	
IQR	5.3–10.7	5.3–10.6	5.3–10.8	5.1–10.3	5.3–10.6	
Current smoker (%)	590 (60.3)	607 (62.1)	593 (60.7)	619 (63.3)	2409 (61.6)	0.516
Alcohol consumption (%)	596 (60.9)	574 (58.7)	573 (58.6)	594 (60.7)	2337 (59.8)	0.589
Physical activity (MET-h/week)						0.021
Median	65.4	60.3	60.0	60.1	60.5	
IQR	13.1–198.9	10.5–168.4	8.9–160.7	10.4–146.2	10.6–170.5	
Antihypertensive medication (%)	59 (6.0)	77 (7.9)	99 (10.1)	147 (15.0)	382 (9.8)	<0.001

Value are expressed as means ± SDs or medians (IQRs) for continuous variables and numbers (percentages, %) for categorical variables. Characteristics of subjects were compared using ANOVA or the Kruskal-Wallis tests for continuous variables and Chi-square for categorical variables. CHNS, the China health and nutrition survey; VAI, visceral adiposity index; BMI, body mass index; WC, waist circumference; SBP, systolic blood pressure; DBP, diastolic blood pressure; FBG, fasting blood glucose; TG, total triglycerides; TC, total cholesterol; HDL-C, high-density lipoprotein cholesterol; LDL-C, low-density lipoprotein cholesterol; SD, standard deviation; IQR, interquartile range; MET, metabolic equivalent.

**Table 2:** Characteristics of the female subjects from the 2009 CHNS according to the quartiles of VAI scores.

Variable	Q1 (< 0.9833)	Q2 (0.9834–1.5854)	Q3 (1.5855–2.7290)	Q4 (≥2.7290)	Total	*p*
No. participants	1120	1122	1121	1120	4483	
Han nationality (%)	998 (89.1)	968 (86.3)	1006 (89.7)	992 (88.6)	3964 (88.4)	0.058
Countryside (%)	771 (68.8)	747 (66.6)	750 (66.9)	734 (65.5)	3002 (67.0)	0.409
Age (years)	45.3 ± 15.3	49.7 ± 15.0	52.5 ± 14.8	54.4 ± 13.4	50.5 ± 15.0	<0.001
BMI (kg/m^2^)	21.8 ± 2.9	22.8 ± 3.4	23.9 ± 2.4	25.2 ± 3.5	23.4 ± 3.5	<0.001
Educational level						<0.001
Middle school or below	835 (74.6)	897 (79.9)	911 (81.3)	935 (83.5)	3578 (79.8)	
High school	207 (18.5)	181 (16.1)	169 (15.1)	158 (14.1)	715 (15.9)	
College or above	78 (7.0)	44 (3.9)	41 (3.7)	27 (2.4)	190 (4.2)	
WC (cm)	75.7 ± 8.7	79.4 ± 9.6	83.1 ± 9.5	87.0 ± 9.7	81.3 ± 10.3	<0.001
SBP (mmHg)	116.4 ± 16.4	122.2 ± 19.5	125.6 ± 20.6	130.6 ± 21.3	123.7± 20.2	<0.001
DBP (mmHg)	75.7 ± 10.3	78.2 ± 11.7	79.6 ± 10.8	82.9 ± 11.6	79.1 ±11.4	<0.001
FBG (mmol/L)	5.0 ± 0.8	5.1 ± 0.8	5.4 ±1.4	5.9 ± 1.8	5.3 ± 1.3	<0.001
TG (mmol/L)	0.7 ± 0.2	1.0 ± 0.2	1.5 ± 0.3	3.0 ± 1.7	1.6 ± 1.2	<0.001
TC (mmol/L)	4.6 ± 0.9	4.8 ± 1.0	5.0 ± 1.0	5.2 ± 1.1	4.9 ± 1.0	<0.001
HDL-C (mmol/L)	1.8 ± 0.6	1.5 ± 0.3	1.4 ± 0.3	1.2 ± 0.3	1.5 ± 0.5	<0.001
LDL-C (mmol/L)	2.8 ± 0.8	3.1 ± 0.9	3.3 ± 0.9	3.1 ± 1.1	3.0 ± 1.0	<0.001
Total energy intake (kcal/day)						
Median	1969.9	1910.7	1902.0	1877.1	1914.0	
IQR	1584.2–2347.9	1564.3–2273.1	1565.2–2262.3	1546.8–2255.3	1567.8–2288.1	
Salt intake (g/day)						
Median	6.2	6.5	6.5	6.4	6.4	
IQR	4.5 – 9.0	4.5 – 9.6	4.6–9.4	4.3–9.2	4.5–9.3	
Current smoker (%)	37 (3.3)	40 (3.6)	45 (4.0)	60 (5.4)	182 (4.1)	0.066
Alcohol consumption (%)	131 (11.7)	93 (8.3)	86 (7.7)	87 (7.8)	397 (8.9)	0.002
Physical activity (MET-h/week)						<0.001
Median	77.9	70.8	60.9	60.3	65.7	
IQR	37.2–156.0	40.5–158.4	35.1–133.6	38.3–115.4	37.9–143.1	
Antihypertensive medication (%)	49 (4.4)	105 (9.4)	128 (11.4)	219 (19.6)	501 (11.2)	<0.001

Value are expressed as means ± SDs or medians (IQRs) for continuous variables and numbers (percentages, %) for categorical variables. Characteristics of subjects were compared using ANOVA or the Kruskal-Wallis tests for continuous variables and Chi-square for categorical variables. CHNS, the China health and nutrition survey; VAI, visceral adiposity index; BMI, body mass index; WC, waist circumference; SBP, systolic blood pressure; DBP, diastolic blood pressure; FBG, fasting blood glucose; TG, total triglycerides; TC, total cholesterol; HDL-C, high-density lipoprotein cholesterol; LDL-C, low-density lipoprotein cholesterol; SD, standard deviation; IQR, interquartile range; MET, metabolic equivalent.

**Table 3:** Associations between the quintile (Q) of VAI scores and blood pressure in the 2009 CHNS. (*n* = 8394).

	Q1	Q2	Q3	Q4	*p*-trend
**SBP**					
**Total**					
β (95% CI) ^1^	Ref	3.296 (2.161, 4.431)	5.742 (4.607, 6.878)	9.632 (8.496, 10.767)	<0.001
Adjusted β (95% CI) ^2^	Ref	2.364 (1.353, 3.375)	4.047 (3.033, 5.062)	7.642 (6.627, 8.658)	<0.001
Adjusted β (95% CI) ^3^	Ref	1.863 (0.890, 2.835)	3.461 (2.484, 5.085)	5.963 (4.977, 6.949)	<0.001
Adjusted β (95% CI) ^4^	Ref	1.663 (0.693, 2.634)	2.974 (1.993, 3.955)	5.197 (4.196, 6.199)	<0.001
**Male**					
β (95% CI) ^1^	Ref	0.341 (-1.212, 1.894)	1.775 (0.222, 3.329)	4.335 (2.781, 5.888)	<0.001
Adjusted β (95% CI) ^5^	Ref	0.928 (-0.492, 2.347)	2.513 (1.087, 3.938)	5.561 (4.132, 6.989)	<0.001
Adjusted β (95% CI) ^6^	Ref	0.563 (-0.814, 1.941)	1.879 (0.493, 3.265)	4.258 (2.858, 5.658)	<0.001
Adjusted β (95% CI) ^7^	Ref	0.320 (-1.053, 1.639)	1.302 (-0.090, 2.694)	3.315 (1.889, 4.740)	<0.001
**Female**					
β (95% CI) ^1^	Ref	5.879 (4.260, 7.498)	9.207 (7.588, 10.826)	14.257 (12.637, 15.877)	<0.001
Adjusted β (95% CI) ^5^	Ref	3.125 (1.690, 6.107)	4.659 (3.212, 6.107)	8.512 (7.051, 9.974)	<0.001
Adjusted β (95% CI) ^6^	Ref	2.533 (1.163, 3.902)	4.221 (2.839, 5.603)	6.627 (5.221, 8.033)	<0.001
Adjusted β (95% CI) ^7^	Ref	2.430 (1.063, 3.798)	3.922 (2.536, 5.308)	6.157 (4.737, 7.577)	<0.001
**DBP**					
**Total**					
β (95% CI) ^1^	Ref	1.708 (1.032, 2.384)	3.134 (2.457, 3.810)	5.927 (5.251, 6.604)	<0.001
Adjusted β (95% CI) ^2^	Ref	1.448 (0.795, 2.100)	2.612 (1.958, 3.267)	5.345 (4.690, 6.000)	<0.001
Adjusted β (95% CI) ^3^	Ref	1.261 (0.623, 1.899)	2.348 (1.707, 2.989)	4.579 (3.931, 5.226)	<0.001
Adjusted β (95% CI) ^4^	Ref	1.105 (0.469, 1.740)	1.966 (1.323, 2.609)	3.978 (3.321, 4.634)	<0.001
**Male**					
β (95% CI) ^1^	Ref	0.881 (-0.904, 1.855)	2.268 (1.293, 3.243)	4.520 (3.545, 5.495)	<0.001
Adjusted β (95% CI) ^5^	Ref	1.036 (0.075, 1.997)	2.441 (1.476, 3.406)	4.858 (3.891, 5.825)	<0.001
Adjusted β (95% CI) ^6^	Ref	0.978 (0.039, 1.918)	2.270 (1.324, 3.216)	4.270 (3.315, 5.226)	<0.001
Adjusted β (95% CI) ^7^	Ref	0.784 (-0.151, 1.719)	1.809 (0.861, 2.757)	3.516 (2.546, 4.487)	<0.001
**Female**					
β (95% CI) ^1^	Ref	2.433 (1.512, 3.354)	3.892 (2.971, 4.814)	7.156 (6.235, 8.078)	<0.001
Adjusted β (95% CI) ^5^	Ref	1.584 (-0.270, 1.347)	2.429 (1.528, 3.330)	5.324 (4.414, 6.234)	<0.001
Adjusted β (95% CI) ^6^	Ref	1.277 (0.403, 2.151)	2.104 (1.222, 2.986)	4.435 (3.538, 5.332)	<0.001
Adjusted β (95% CI) ^7^	Ref	1.193 (0.321, 2.065)	1.859 (0.975, 2.742)	4.409 (3.144, 4.954)	<0.001

Q, quintile; VAI, visceral adiposity index; SBP, systolic blood pressure; DBP, diastolic blood pressure; CI, confidence interval; Ref, reference; MET-h, metabolic equivalent-hour; TC, total cholesterol. ^1^Model 1: original model without any adjustments; ^2^Model 2: adjusted for countryside, age, gender, nationality (Han or others), education (middle school or below, high school, or college or above); ^3^Model 3: adjusted as for model 2 plus smoking status (current or not current), alcohol consumption (yes or no), physical activity (unknown, 0.0–46.0, 46.1–97.8, 97.9–187.9, or ≥188.0 MET-h/week), antihypertensive medication (yes or no), total energy intake, and salt intake; ^4^Model 4: adjusted as for model 3 plus TC; ^5^Model 5: adjusted as for model 2 minus gender; ^6^Model 6: adjusted as for model 3 minus gender; ^7^Model 7: adjusted as for model 4 minus gender.

**Table 4:** Multivariable-adjusted odds ratios (and 95% CIs) of hypertension according to quintile (Q) of VAI scores in the 2009 CHNS (*n* = 8394)**.**

	Q1	Q2	Q3	Q4	*p*-trend
**Total**					
OR (95% CI) ^1^	Ref	1.460 (1.266, 1.683)	1.827 (1.588, 2.101)	2.903 (2.533, 3.327)	<0.001
Adjusted OR (95% CI) ^2^	Ref	1.457 (1.248, 1.700)	1.749 (1.502, 2.036)	2.944 (2.537, 3.417)	<0.001
Adjusted OR (95% CI) ^3^	Ref	1.331 (1.121, 1.581)	1.614 (1.362, 1.911)	2.454 (2.076, 2.901)	<0.001
Adjusted OR (95% CI) ^4^	Ref	1.308 (1.101, 1.554)	1.546 (1.304, 1.834)	2.299 (1.939, 2.726)	<0.001
**Male**					
OR (95% CI) ^1^	Ref	1.078 (0.886, 1.311)	1.293 (1.066, 1.567)	1.910 (1.582, 2.306)	<0.001
Adjusted OR (95% CI) ^5^	Ref	1.175 (0.953, 1.447)	1.472 (1.197, 1.811)	2.439 (1.988, 2.994)	<0.001
Adjusted OR (95% CI) ^6^	Ref	1.094 (0.871, 1.373)	1.321 (1.052, 1.658)	2.036 (1.625, 2.550)	<0.001
Adjusted OR (95% CI) ^7^	Ref	1.067 (0.849, 1.340)	1.245 (0.989, 1.566)	1.849 (1.467, 2.329)	<0.001
**Female**					
OR (95% CI) ^1^	Ref	2.091 (1.688, 2.591)	2.724 (2.209, 3.359)	4.616 (3.761, 5.666)	<0.001
Adjusted OR (95% CI) ^5^	Ref	1.781 (1.409, 2.252)	2.004 (1.593, 2.521)	3.350 (2.694, 4.221)	<0.001
Adjusted OR (95% CI) ^6^	Ref	1.640 (1.253, 2.146)	1.925 (1.481, 2.502)	2.847 (2.198, 3.689)	<0.001
Adjusted OR (95% CI) ^7^	Ref	1.631 (1.247, 2.135)	1.896 (1.457, 2.467)	2.781 (2.141, 3.612)	<0.001

CI, confidence interval; Q, quintile; VAI, visceral adiposity index; CHNS, the China Health and Nutrition Survey; OR, odds ratio; Ref, reference; MET-h, metabolic equivalent-hour; TC, total cholesterol. ^1^Model 1: original model without any adjustments; ^2^Model 2: adjusted for countryside, age, gender, nationality (Han or others), education (middle school or below, high school, or college or above); ^3^Model 3: adjusted as for model 2 plus smoking status (current or not current), alcohol consumption (yes or no), physical activity (unknown, 0.0–46.0, 46.1–97.8, 97.9–187.9, or ≥ 188.0 MET-h/week), antihypertensive medication (yes or no), total energy intake, and salt intake; ^4^Model 4: adjusted as for model 3 plus TC; ^5^Model 5: adjusted as for model 2 minus gender; ^6^Model 6: adjusted as for model 3 minus gender; ^7^Model 7: adjusted as for model 4 minus gender.

**Table 5:** Multivariable-adjusted hazard ratios (and 95% CIs) of hypertension according to quintile (Q) of VAI scores in a follow up from 2009 to 2011 (*n* = 4275).

	Q1	Q2	Q3	Q4	*p*-trend
**Total**					
Patient / total participants	117 / 1198	154 / 1122	147/1052	169/903	
Person-years	1.97 ± 0.08	1.97 ± 0.08	1.98 ± 0.08	1.97 ± 0.08	
Crude HR (95% CI) ^1^	Ref	1.398 (1.099, 1.777)	1.341 (1.052, 1.710)	1.692 (1.337, 2.143)	<0.001
Adjusted HR (95% CI) ^2^	Ref	1.302 (1.023, 1.658)	1.218 (0.955, 1.554)	1.624 (1.282, 2.057)	0.001
Adjusted HR (95% CI) ^3^	Ref	1.306 (1.024, 1.665)	1.193 (0.932, 1.526)	1.641 (1.292, 2.083)	0.001
Adjusted HR (95% CI) ^4^	Ref	1.283 (1.006, 1.637)	1.141 (0.890, 1.464)	1.526 (1.194, 1.952)	0.005
**Male**					
Patient / total participants	64 / 492	85 / 500	66 / 471	88 / 413	
Person-years	1.97 ± 0.08	1.97 ± 0.08	1.98 ± 0.08	1.98 ± 0.08	
Crude HR (95% CI) ^1^	Ref	1.219 (0.881, 1.687)	0.885 (0.627, 1.251)	1.367 (0.990, 1.887)	0.036
Adjusted HR (95% CI) ^5^	Ref	1.200 (0.864, 1.665)	0.948 (0.670, 1.342)	1.600 (1.154, 2.219)	0.005
Adjusted HR (95% CI) ^6^	Ref	1.195 (0.858, 1.663)	0.926 (0.653, 1.315)	1.601 (1.152, 2.224)	0.004
Adjusted HR (95% CI) ^7^	Ref	1.171 (0.841, 1.632)	0.889 (0.624, 1.266)	1.497 (1.067, 2.101)	0.010
**Female**					
Patient / total participants	53 / 1198	69 / 622	81 / 581	81 / 490	
Person-years	1.98 ± 0.08	1.98 ± 0.08	1.97 ± 0.08	1.98 ± 0.08	
Crude HR (95% CI) ^1^	Ref	1.537 (1.074, 2.199)	1.915 (1.355, 2.708)	2.025 (1.432, 2.863)	<0.001
Adjusted HR (95% CI) ^5^	Ref	1.379 (0.963, 1.976)	1.462 (1.032, 2.072)	1.497 (1.055, 2.123)	0.108
Adjusted HR (95% CI) ^6^	Ref	1.469 (1.020, 2.115)	1.463 (1.025, 2.088)	1.574 (1.102, 2.246)	0.071
Adjusted HR (95% CI) ^7^	Ref	1.457 (1.011, 2.099)	1.418 (0.990, 2.031)	1.498 (1.041, 2.157)	0.124

CI, confidence interval; Q, quintile; VAI, visceral adiposity index; HR, hazard ratio; Ref, reference; MET-h, metabolic equivalent-hour; TC, total cholesterol. ^1^Model 1: original model without any adjustments; ^2^Model 2: adjusted for countryside, age, gender, nationality (Han or others), education (middle school or below, high school, or college or above); ^3^Model 3: adjusted as for model 2 plus smoking status (current or not current), alcohol consumption (yes or no), physical activity (unknown, 0.0–46.0, 46.1–97.8, 97.9–187.9, or ≥188.0 MET-h/week), antihypertensive medication (yes or no), total energy intake, and salt intake; ^4^Model 4: adjusted as for model 3 plus TC; ^5^Model 5: adjusted as for model 2 minus gender; ^6^Model 6: adjusted as for model 3 minus gender; ^7^Model 7: adjusted as for model 4 minus gender.
